# Transcript analysis of some defense genes of tomato in response to host and non-host bacterial pathogens

**DOI:** 10.22099/mbrc.2017.25600.1273

**Published:** 2017-12

**Authors:** Ali Safaie-Farahani, S. Mohsen Taghavi

**Affiliations:** Department of Plant Protection, College of Agriculture, Shiraz University, Shiraz, Iran

**Keywords:** PR-1, PR-2, PR-5, LOX, PAL, CAT

## Abstract

The transcript levels of six defense genes including pathogenesis-related gene 1 (*PR-1*), pathogenesis-related gene 2 (*PR-2*), pathogenesis-related gene 5 (*PR-5*), lipoxygenase (*LOX*), phenylalanine ammonia-lyase (*PAL*) and catalase (*CAT*) were investigated in tomato plants inoculated with *Xanthomonas axonopodis* pv. *phaseoli* as a non-host pathogen and *X. euvesicatoria *as a host pathogen. Activation of all the genes was confirmed in both host and non-host treatments. Additionally, the results showed stronger expression of majority of the genes (*PR-1*, *PR-2*, *LOX*, *PAL* and *CAT*) in non-host treatment compared to host treatment at least at early hours after inoculation. These data suggest that faster and more expression of *PR-1*, *PR-2*, *LOX*, *PAL* and *CAT* might have a role in non-host resistance of tomato against *X. axonopodis* pv. *phaseoli*.

## INTRODUCTION

Plants have ability to protect themselves against pathogen attack by numerous strategies. Production of pathogenesis-related (PR) proteins in plants is an important defense mechanism versus pathogen invasion. Most PR proteins are acid-soluble, low molecular weight and protease-resistant proteins. Based on their sequences and functions, PR-proteins have been divided into 17 families [1]. Lipoxygenase (LOX) are a group of nonheme iron-containing dioxygenases that initiate the degradation of free fatty acids and esterified lipids via various branches of the LOX pathway. LOX may act as a signaling molecule which be involved in structural and metabolic changes in plant leading resistance to pathogen [2]. LOX activation in plants in response to environmental and biotic stresses has been reported [3-5]. Phenylalanine ammonia lyase (PAL) is a key enzyme of the phenylpropanoid pathway that catalyzes the deamination of phenylalanine to cinnamic acid, a precursor for the lignin and flavonoid biosynthetic pathways [6]. Induction of PAL in plants infected with pathogens has been shown [7, 8]. Superoxide, hydrogen peroxide and hydroxyl radical are various types of reactive oxygen species (ROS) which might be produced in plants upon pathogen infection. On the other hand, antioxidant enzymes such as superoxide dismutase (SOD), ascorbate peroxidase (APX) and catalase (CAT) can be employed by plants to avoid the harmful effects of ROS [9]. 

Non-host resistance is a resistance displayed by a whole plant species versus all genetic variants of a non-adapted pathogen species. It is a long-lasting and robust resistance against numerous pathogens. Non-host resistance is divided into two types, based on presence or absence of visual symptoms. Type I is not associated with any visual symptoms, while type II produce visual necrosis spots [10]. Despite recent advances in clarification of the molecular aspects of non-host resistance against plant pathogens, molecular mechanisms underpinning non-host resistance remain relatively unexplored [11]. Hence, the goal of this study was to survey the transcript abundances of some defense genes of tomato including *PR-1*, *PR-2*, *PR-5*, *LOX*, *PAL* and *CAT* in response to *Xanthomonas axonopodis* pv. *phaseoli*, as a non-host pathogen. Furthermore, transcript abundances of the genes were investigated during tomato infection by *X. euvesicatoria*, the causal agent of bacterial spot.

## MATERIALS AND METHODS

**Plant materials and pathogen treatments:** Tomato (*Solanum lycopersicum* cv. Early orbano) seeds were surface-sterilized by 1.0% sodium hypochlorite (20% household bleach) for 5 min and then sown in quartz sand in 10-cm plastic pots in a growth chamber. *X. euvesicatoria* Xeu3 [12] and *X. axonopodis* pv. *phaseoli *K1 [13] were used as a host and non-host pathogens, respectively. Bacterial inocula were provided in sterile distilled water at a concentration of about 10^8^ CFU/ml and were sprayed on the leaves of six-week-old plants. Sterile distilled water was used as a negative control. Plants were incubated at 28±1.0^o^C with 16-h light daily and 70% relative humidity. The leaves were harvested at 12, 24, 48 and 72 hours post pathogen inoculation (hpi) separately, frozen in liquid nitrogen immediately then stored in -80^o^C. 

**RNA extraction, c-DNA synthesis and** **Real-time RT-PCR reaction** **:** Total RNA was isolated using an extraction kit (DENAzist, Iran), according to the manufacturer’s protocol. For each sample, RNA concentration was determined using a spectrophoto-meter, and the samples with a 260:280 ratio between 1.9 and 2.1 were used for the analysis. Agarose gel electrophoresis was also employed to approve the RNA integrity of each sample. Isolated RNA was treated with DNase I (Fermentas, Lithuania) and then subjected to reverse transcription reaction using a commercial kit (Fermentas, Lithuania) according to the manufacturer’s instruction. The cDNA samples were diluted into 1:10 ratio with sterile double distill water and stored at -80°C before being used as template in real-time PCR. Real-time RT-PCR was performed in a thermocycler (Bioneer, South Korea) using the following scheme: 5 min at 94^o^C, followed by 40 cycles of 1 min at 94^o^C, 1 min at 58^o^C and 1 min at 72^o^C, with final extension for 10 min at 72^o^C. The expression detected from *actin* and *β-tubulin* genes was used as internal reference. The primers used in this study are listed in Table 1. The changes of transcript concentration were measured by the comparative 2^−ΔΔCT^ technique [14]. The experiments were repeated three times for each sample and the results were averaged. The data were assessed by analysis of variance (ANOVA) using SAS 9.1 (SAS Institute, Cary, NC, USA). The means were separated by Duncan’s multiple range tests.

**Table 1:** The primers used in this study.

**Target gene**	**Forward sequence**	**Reverse sequence**	**reference**
*PR-1*	GGATCGGACAACGTCCTTAC	GCAACATCAAAAGGGAAATAAT	[15]
*PR-2*	AAGTATATAGCTGTTGGTAATGAA	ATTCTCATCAAACATGGCGAA	[15]
*PR-5*	GAGGTTCATGCCAAACTGGTC	CCGTCAACCAAAGAAATGTCC	[16]
*LOX*	GGCTTGCTTTACTCCTGGTC	AAATCAAAGCGCCAGTTCTT	[17]
*PAL*	ACGGGTTGCCATCTAATCTG	AGCTCTTTTCCTGGCTGAAA	[18]
*CAT*	TGGAAGCCAACTTGTGGTGT	ACTGGGATCAACGGCAAGAG	[19]
*Actin*	AACTGGGATGATATGGAGAAGA	TCTCAACATAATCTGGGTCAT	[17]
*β-tubulin*	AACCTCCATTCAGGAGATGTTT	TCTGCTGTAGCATCCTGGTATT	[18]

## RESULTS

Irregular dark spots surrounded by chlorotic halos were observed on tomato leaves inoculated with *X. euvesicatoria* within 13-18 days post inoculation. In contrast, no symptoms were found in tomato plants inoculated with *X. axonopodis* pv. *phaseoli*.

*PR-1* transcript in non-host treatment was significantly higher compared to host treatment at 12 and 24 hpi. There was no significant difference between host and non-host treatments in terms of *PR-1* transcript at 48 hpi. On the other hand, *PR-1* transcript in host treatment was significantly higher than non-host treatment at 72 hpi. *PR-2* transcript in non-host treatment was significantly higher compared to host treatment at 12 and 24 hpi. There was no significant difference between host and non-host treatments in terms of *PR-2* transcript at 48 and 72 hpi. *PR-5* transcript in host treatment was significantly higher than non-host treatment at all time points. *LOX* transcript in non-host treatment was significantly higher compared to host treatment at 12, 24 and 48 hpi. On the other hand, *LOX* transcript in non-host treatment was significantly lower than host treatment at 72 hpi. *PAL* transcript in non-host treatment was significantly higher compared to host treatment at 12 and 24 hpi. There was no significant difference between host and non-host treatments in terms of *PAL* transcript at 48 hpi. *PAL* transcript in non-host treatment was significantly lower than host treatment at 72 hpi. *CAT* transcript in non-host treatment was significantly higher compared to host treatment at 12 and 24 hpi. On the other hand, *CAT* transcript in host treatment was significantly higher than non-host treatment at 48 and 72 hpi (Table 2).

**Table 2:** Fold-changes (±SD) in transcript levels of *PR-1*, *PR-2*, *PR-5*, *LOX*, *PAL* and *CAT* in non-host treatment (left numbers) and host treatment (right numbers) compared to control

**Genes**	**Hours post pathogen inoculation (hpi)**			
	**12**	**24**	**48**	**72**
*PR-1*	4.3±0.32a/ /1.3±0.07b	7.2±0.77a/2.1±0.14b	3.7±0.42a/3.5±0.29a	2.4±0.18b/4.1±0.49a
*PR-2*	6.3±0.72a/2.7±0.17b	8.2±0.96a/3.7±0.40b	4.7±0.38a/4.5±0.65a	2.9±0.13a/3.0±0.36a
*PR-5*	2.5±0.18b/4.9±0.81a	2.1±0.12b/3.7±0.39a	1.8±0.21b/3.5±0.41a	1.1±0.06b/1.9±0.10a
*LOX*	3.6±0.52a/1.7±0.13b	4.7±0.68a/2.9±0.18b	6.9±0.50a/4.1±0.29b	4.2±0.71b/6.5±0.63a
*PAL*	2.1±0.15a/1.3±0.08b	3.9±0.35a/2.3±0.27b	2.4±0.14a/2.5±0.20a	1.8±0.22b/2.8±0.16a
*CAT*	5.9±1.03a/1.3±0.14b	4.8±0.81a/2.6±0.26b	2.2±0.41b/5.4±1.09a	1.3±0.07b/3.3±0.39a

In each time point, means with diverse letters are significantly different at p<0.05.

## DISCUSSION

Understanding of non-host resistance mechanisms is imperative to engineer cultivars in plant breeding programs. This study was performed to elucidate whether or not tomato plants susceptible to bacterial spot display similar defense responses after inoculation with the non-host pathogen. Transcript changes of six defense genes including *PR-1* (unknown function), *PR-2* (β-1,3-glucanase), *PR-5* (osmotin), *LOX*, *PAL* and *CAT* was compared between host and non-host treatments. Our results showed that expression of majority of the genes in non-host treatment is significantly higher compared to host treatment at least at early stages after inoculation. Therefore, it can be speculated that faster and stronger expression of the genes play important role in non-host resistance. On the other hand, more expression of *PR-5* in host treatment than non-host treatment showing that molecular mechanism of non-host resistance is complex. Some overlaps between plants responses to host and non-host pathogens suggesting that plants may recognize similar factors in both host and non-host pathogens for initiating defense responses [20]. For instance, the harpin elicitor of *Pseudomonas syringae* pv. *phaseolicola* is recognized by the non-host plant, tobacco, and stimulate defense responses such as induction of *PR* genes [21]. Accumulation of *PR-1*, *PR-2* and *PR-5* transcripts has been found in broad bean plants inoculated with *Puccinia striiformis* f. sp. *tritici*, a non-host pathogen [22]. In grapevine, expression of some defense genes such as *PR-2* is affected by a non-host pathogen, *Pseudoperonospora* *cubensis* [23]. Glucan production (following activity of β-1,3-glucanase) might motivate induction of other defense responses such as phytoalexin production [24] and PAL induction [25]. The role of osmotin in plant cells protection from osmotic shock through structural or metabolic modifications is proved [26]. Earlier and more expression of *LOX* in cucumber in response to *P.* *syringae* pv. *syringae*, a non-host pathogen, compared to *P. syringae* pv. *lachrymans*, a host pathogen, is observed [5]. The role of LOX in plant defense against biotic stresses seems to be related to the synthesis of various compounds with signaling functions [27]. PAL protein is demonstrated to accumulate in *Arabidopsis *plants inoculated with two non-host bacteria, *P.* *syringae* pv. *phaseolicola* and *P.* *syringae* pv. *glycinea* (28). Additionally, *PAL Arabidopsis* mutants show more growth of these non-host pathogens, compared to the wild-type plants [28]. Regarding to cinnamic acid is the precursor of numerous secondary metabolites [6], faster and stronger expression of PAL is vital for plant resistance. Production of ROS is one of the earliest defense responses in plant versus pathogen invasion [9]. Accumulation of ROS plays important role in some non-host interactions such as barley/*Blumeria graminis *f. sp. *tritici *[29], cowpea/*Erysiphe cichoracearum *[30], pepper/*Blumeria graminis *f. sp. *tritici* interactions [31] and tomato/*Magnaporthe grisea* [32]. Increased induction of ROS during non-host interaction can restrict further growth of pathogen in plant. On the other hand, balanced amounts of ROS (as a consequence of antioxidant enzymes activity) could act as an inducer of other defense responses [33]. Therefore, earlier expression of antioxidant enzymes might have a major role in induction of other defense mechanisms. In mung bean, more activity of antioxidant enzymes including CAT is found in incompatible interaction (mung bean/*X. hortorum* pv. *pelargonii*) rather than compatible interaction (mung bean/*X. axonopodis* pv. *phaseoli*) [34]. In conclusion, different expression of *PR-1*, *PR-2*, *PR-5*, *LOX*, *PAL* and *CAT* in response to host and non-host bacterial pathogens was confirmed in this study. These finding might be considered in plant breeding programs. 
